# Wheat Stem Rust Back in Europe: Diversity, Prevalence and Impact on Host Resistance

**DOI:** 10.3389/fpls.2022.882440

**Published:** 2022-06-02

**Authors:** Mehran Patpour, Mogens S. Hovmøller, Julian Rodriguez-Algaba, Biagio Randazzo, Dolors Villegas, Vladimir P. Shamanin, Anna Berlin, Kerstin Flath, Pawel Czembor, Alena Hanzalova, Svetlana Sliková, Ekaterina S. Skolotneva, Yue Jin, Les Szabo, Kevin J. G. Meyer, Romain Valade, Tine Thach, Jens G. Hansen, Annemarie F. Justesen

**Affiliations:** ^1^Department of Agroecology, Aarhus University, Slagelse, Denmark; ^2^Società Semplice Agricola Randazzo (AS.A.R.), Palermo, Italy; ^3^Institute for Food and Agricultural Research and Technology (IRTA), Lleida, Spain; ^4^Department of Agronomy, Omsk State Agrarian University, Omsk, Russia; ^5^Department of Forest Mycology and Plant Pathology, Swedish University of Agricultural Science, Uppsala, Sweden; ^6^Julius Kühn-Institut, Federal Research Centre for Cultivated Plants, Institute for Plant Protection in Field Crops and Grassland, Quedlinburg, Germany; ^7^Plant Breeding & Acclimatization Institute – National Research Institute, Radzików, Poland; ^8^Department of Genetics and Plant Breeding Methods, Crop Research Institute, Prague, Czechia; ^9^National Agricultural and Food Centre, Nitra, Slovakia; ^10^Institute of Cytology and Genetics, Russian Academy of Sciences, Novosibirsk, Russia; ^11^USDA-ARS Cereal Disease Laboratory, University of Minnesota, Minneapolis, MN, United States; ^12^Université Paris-Saclay, INRAE, UR BIOGER Thiverval-Grignon, France; ^13^ARVALIS Institut du Végétal, Boigneville, France

**Keywords:** *Puccinia graminis*, black rust, re-emergence, exotic incursion, common barberry, virulence, *Sr31*

## Abstract

The objective of this study was to investigate the re-emergence of a previously important crop pathogen in Europe, *Puccinia graminis* f.sp. *tritici,* causing wheat stem rust. The pathogen has been insignificant in Europe for more than 60 years, but since 2016 it has caused epidemics on both durum wheat and bread wheat in local areas in southern Europe, and additional outbreaks in Central- and West Europe. The prevalence of three distinct genotypes/races in many areas, Clade III-B (TTRTF), Clade IV-B (TKTTF) and Clade IV-F (TKKTF), suggested clonal reproduction and evolution by mutation within these. None of these genetic groups and races, which likely originated from exotic incursions, were detected in Europe prior to 2016. A fourth genetic group, Clade VIII, detected in Germany (2013), was observed in several years in Central- and East Europe. Tests of representative European wheat varieties with prevalent races revealed high level of susceptibility. In contrast, high diversity with respect to virulence and Simple Sequence Repeat (SSR) markers were detected in local populations on cereals and grasses in proximity to *Berberis* species in Spain and Sweden, indicating that the alternate host may return as functional component of the epidemiology of wheat stem rust in Europe. A geographically distant population from Omsk and Novosibirsk in western Siberia (Russia) also revealed high genetic diversity, but clearly different from current European populations. The presence of *Sr31*-virulence in multiple and highly diverse races in local populations in Spain and Siberia stress that virulence may emerge independently when large geographical areas and time spans are considered and that *Sr31*-virulence is not unique to Ug99. All isolates of the Spanish populations, collected from wheat, rye and grass species, were succesfully recovered on wheat, which underline the plasticity of host barriers within *P. graminis*. The study demonstrated successful alignment of two genotyping approaches and race phenotyping methodologies employed by different laboratories, which also allowed us to line up with previous European and international studies of wheat stem rust. Our results suggest new initiatives within disease surveillance, epidemiological research and resistance breeding to meet current and future challenges by wheat stem rust in Europe and beyond.

## Introduction

Stem rust on wheat, caused by the basidiomycete *Puccinia graminis* f.sp. *tritici* (*Pgt*) Eriks. & E. Henn., has historically been considered a main threat for wheat production globally due to the risks of high losses in grain yield and quality (Singh et al., 2015). The disease, which is also known as black rust, became less prevalent in Europe during the second half of the twentieth century, due to a combination of efforts to eradicate common barberry (*Berberis vulgaris*), the alternate host for multiple *formae speciales* of *Pg* (Lind, 1915; Stakman, 1923; Hermansen, 1968), as well as advances in plant breeding and plant disease management. Nevertheless, stem rust persisted as a potential threat mainly in warmer wheat growing areas in Central and Eastern Europe (Bartoš et al., 1996). In Africa, the situation has changed since 1999 by the emergence of a new race group, termed ‘Ug99,’ which carries virulence to stem rust resistance gene *Sr31,* thereby leaving many wheat cultivars susceptible to stem rust (Pretorius et al., 2000; Singh et al., 2011). Since then, stem rust of different genetic groups and races have emerged in East Africa, for example race TKTTF, which resulted in epidemics in Ethiopia in 2013 with yield losses close to 100% in the most grown wheat cultivar., ‘Digalu’ (Olivera et al., 2015).

In 2013, stem rust reappeared on several wheat cultivars at several locations in Germany (Olivera Firpo et al., 2017) along with sporadic incidences in southern Denmark, eastern Sweden and the United Kingdom (Lewis et al., 2018). A more widespread outbreak was reported from Sicily in Italy in 2016, where thousands of hectares of both bread and durum wheat were affected (Bhattacharya, 2017). Preliminary analyses of infected plant samples from Sicily suggested the presence of a new race in Europe, TTRTF (termed TTTTF in Bhattacharya (2017)), which carried virulence to *Sr13b* of particular relevance for durum wheat (Patpour et al., 2020). These events were followed by the detection of stem rust on late maturing wheat and barley in local areas in Sweden in 2017, characterized by cool temperatures and an unusual wet growing season. Since then, stem rust has been observed every year on wheat, barley and rye in these areas (Kjellström, 2021). Earlier studies in Sweden have shown that stem rust on oat and rye reproduce asexually on the cereal host and sexually on the alternate host, *B. vulgaris* (Berlin et al., 2012).

A new initiative to establish a European early-warning system for wheat rust diseases, RustWatch, was launched in May 2018, including the coordination of national wheat rust surveillance and rust resistance breeding efforts.^1^ This initiative builds on the achievements of the Global Rust Reference Center (GRRC) at Aarhus University, Denmark, and the Borlaug Global Rust Initiative, which was launched in 2008 in response to the emergence of the stem rust race Ug99 and multiple variants (McIntosh and Pretorius, 2011). The role of the alternate host in the epidemiology of *Pgt* in Europe at present time was an important part of the RustWatch initiative, following the increase in prevalence in at least Sweden and the United Kingdom, after the lapse in legislation about planting and eradication of common barberry in the 1990s (Berlin et al., 2012; Saunders et al., 2019).

In this study, we investigated whether stem rust outbreaks in Europe since 2016 could be connected to a local outbreak in Germany in 2013, and to which extent the populations were influenced by exotic incursions from outside Europe as opposed to evolution by mutation and/or sexual recombination in local populations in proximity to *Berberis* spp. These hypotheses were explored by extensive sampling of stem rust from new outbreaks in agricultural areas in Europe as well as in local areas in Sweden and Spain, where the alternate host has been documented, and from a distant wheat growing area in western Siberia, Russia, where more than 1 million hectares of wheat were affected by stem rust epidemics in 2015 with yield losses of up to 20–30% (Shamanin et al., 2016). All samples were genotyped to investigate genetic variability and relatedness, and subsets were race typed to investigate phenotypic diversity for virulence and epidemic potential on European wheat varieties.

## Materials and Methods

### Collection and Recovery of Wheat Stem Rust Samples

A total of 479 samples of stem rust from mainly wheat, occasionally other cereals, and grasses, were collected from 17 European countries and western Siberia, Russia (Table 1). The number of samples varied across years and locations, largely reflecting sporadic and variable occurrence and sampling efforts of wheat stem rust.

**Table 1:** Number of dead and recovered stem rust samples collected between 2016 and 2021.

Country	Sampling year	Sample status		
		Dead	Recovered	Total
Austria	2020, 2021	0	5	5
Belgium	2021	1	1	2
Croatia	2017	9	2	11
Czech Republic	2018, 2020, 2021	1	11	12
Denmark	2018–2021	2	15	17
France	2019, 2020, 2021	14	28	42
Germany	2019, 2020	0	4	4
Hungary	2019, 2020	1	2	3
Ireland	2020	1	1	2
Italy	2016–2021	35	166	201
Norway	2020, 2021	0	3	3
Poland	2019, 2020	1	8	9
Russia	2016, 2017	6	38	44
Slovak Republic	2018, 2020, 2021	2	18	20
Slovenia	2020, 2021	5	9	14
Spain	2018–2021	3	34	37
Sweden	2017–2019, 2021	10	33	43
Switzerland	2021	0	10	10
Total		91	388	479

Twenty-five reference isolates of diverse geographical origin, including isolates representing the German outbreak in 2013, and DNA samples hosted by the USDA ARS Cereal Disease Lab, Minnesota, United States, were included to ensure alignment with previous studies and genotyping methodologies (Olivera Firpo et al., 2017; Lewis et al., 2018; Szabo et al., 2022). Live isolates were exchanged under strict quarantine conditions, and DNA samples representing all previously defined clades/genetic groups in *Pgt* were tested in duplicate using SNP and SSR genotyping approaches (Table 2). Mailing and shipment of samples from the field followed the protocol by (Olivera et al., 2015). Most of the samples from western Siberia were collected during a surveillance trip in 2016 and additional samples from the same areas were submitted to GRRC in 2017. Samples from Sweden were part of a comprehensive stem rust survey in 2018–2019, following the first detection of a sexual *Pgt* population on barley and wheat in proximity to common barberry in the autumn of 2017. Samples from Spain were collected from cereal and grass hosts in areas in close proximity to indigenous subspecies of the alternate host, *B. vulgaris*, and in contrasting areas where the alternate host was not present. Samples from France, 2021, were part of a national stem rust survey initiated as an early reaction to the widespread appearance of the disease in June–July 2021.

**Table 2:** Reference isolates of diverse origin, including isolates from the outbreak in Germany in 2013 to facilitate alignment of race, SSR genotype and SNP genotype in *Puccinia graminis* f.sp. *tritici.*

Genetic group/Clade	Ref. isolate	Provided by^a^	Geographical origin	Genotyping method	References	Confirmed race
Clade I	07KEN24-4	CDL	East Africa	SNP chip & SSR	Szabo et al., 2022	TTTSK
Clade I	ET165-12	GRRC	East Africa	SNP chip & SSR	Current paper	TTKSK
Clade II	13ETH25-2	CDL	East Africa	SNP chip & SSR	Olivera et al., 2015	JRCQC
Clade II	ET45a1–14	GRRC	East Africa	SNP chip & SSR	Current paper	JRCQC
Clade III-A	06YEM34-1	CDL	Middle East	SNP chip & SSR	Szabo et al., 2022	TRTTF
Clade III-B	14GEO189-1	CDL	West Asia	SNP chip & SSR	Olivera et al., 2019	TTRTF
Clade III-B	IT14a1–16	GRRC	Europe	SNP core set & SSR	Current paper	TTRTF
Clade IV-A.1	13GER17-3	CDL	Germany (2013)	SNP chip & SSR	Olivera Firpo et al., 2017	TKTTF
Clade IV-A.1	DE205a-13	GRRC	Germany (2013)	SNP chip & SSR	Current paper	TKTTF
Clade IV-A.2	13GER10-4	CDL	Germany (2013)	SNP chip & SSR	Olivera Firpo et al., 2017; Szabo et al., 2022	TKTTF
Clade IV-A.2	DK185a-13	GRRC	Europe	SNP chip & SSR	Current paper	TKTTF
Clade IV-B	TZ92a-16	GRRC	East Africa	SNP core set & SSR	Current paper	TTTTF
Clade IV-B	ET320a-15	GRRC	East Africa	SNP chip & SSR	Current paper	TKTTF
Clade IV-C	13GER06-1	CDL	Germany (2013)	SNP chip & SSR	Olivera Firpo et al., 2017; Szabo et al., 2022	PKPTF
Clade IV-C	AZ180a-14	GRRC	West Asia	SNP chip & SSR	Current paper	PKTTF
Clade IV-D	13GER01-1	CDL	Germany (2013)	SNP chip & SSR	Olivera Firpo et al., 2017; Szabo et al., 2022	TKKTF
Clade IV-E.1	13GER16-4	CDL	Germany (2013)	SNP chip & SSR	Olivera Firpo et al., 2017; Szabo et al., 2022	TKKTP
Clade IV-E.1	DEWST62-13-10	GRRC	Germany (2013)	SSR	Current paper	TKKTP
Clade IV-E.2	13GER08-1	CDL	Germany (2013)	SNP chip & SSR	Olivera Firpo et al., 2017	TKKTF
Clade IV-E.2	SE373a1–14	GRRC	Europe	SNP chip & SSR	Current paper	TKKTF
Clade IV-F	14GEO190-2	CDL	West Asia	SNP chip & SSR	Olivera et al., 2019; Szabo et al., 2022	TKFTF
Clade IV-F	IT33a-18	GRRC	Europe	SSR	Current paper	TKKTF
Clade VI-C	75–36–700-3 (SZA7a)	CDL	North America	SNP chip & SSR	Szabo et al., 2022	SCCLC
Clade VIII	WST55-13-6	JKI	Germany (2013)	SSR	Current paper	HFCNC
-	WSR67-13-5	JKI	Germany (2013)	SSR	Current paper	MMMTF

a *USDA-ARS Cereal Disease Laboratory, University of Minnesota, United States, GRRC: Global Rust Reference Center, Aarhus University, Denmark, JKI: Julius Kühn-Institut, Dresden, Germany*.

Recovery was facilitated by placing stem and/or leaves on moist filter papers in petri dishes under humid conditions at 18°C for 1–2 days to promote the formation of urediniospores. Thereafter, the infected tissue of each sample was gently rubbed on the adaxial side of seedling leaves of the universally stem rust susceptible wheat lines Morocco, Line E and McNair 701. The seedlings were grown in pots, 12–15 plants per pot and treated with 5 ml of 0.5% Antergon MH180 (Nordisk Alkali, Randers, Denmark) to regulate plant growth and enhance spore production. The inoculated seedlings were misted with water and incubated in a dew chamber at 18°C in darkness for 24 h under high relative humidity (RH), after which they were transferred to spore-proof greenhouse cabins set to 20°C during day/18°C during night with a 16 h photoperiod of natural light and supplemental sodium light (200 μmol s^−1^ m^−2^) and 8 h darkness. Plants were watered automatically with added micronutrients for 10 min every 12 h. The pots were covered with cellophane bags (Helmut Schmidt Verpackungsfolien GmbH, Königswinter, Germany) before onset of sporulation to avoid cross contamination between samples. Urediniospores from each sample were harvested, dried, and stored in a liquid nitrogen cryo-container (−196°C), or freezer (−80°C), until further use.

### DNA Extraction

Genomic DNA was extracted from leaf segments representing 459 samples, typically 1–2 cm, containing single pustules of *Pgt* taken from multiplication pots or directly from infected stem segments of incoming samples. The specimens were dried and stored in a desiccator for 24–48 h. Prior to DNA extraction, each specimen was ground with one steel ball (ø 4 mm) and equal amounts of acid washed sand in a 2 ml Eppendorf tube at 1500× strikes for 180 s using a Geno/Grinder 2010 (SPEX SamplePrep, United States). The powdered material was suspended in 300 μl lysis buffer PN and 1 μl RNaseA (100 mg/ml) from the Sbeadex^®^ Mini Plant Kit (LGC Genomics, Germany). The lysate was incubated at 65°C for 30 min followed by centrifugation at 10,000 rpm for 10 min. 50 μl of the supernatant was used for DNA extraction, automated with a KingFisher^™^ Magnetic Particle Processor (Thermo Fisher Scientific, United States) according to manufacturer instructions for the Sbeadex^®^ Mini Plant Kit (LGC Genomics, Germany). DNA was eluted in 50 μl of elution buffer provided by the manufacturer.

### SSR Genotyping

Seventeen Simple Sequence Repeat (SSR) primer pairs previously applied by Stoxen (2012), Jin et al. (2009), and Zhong et al. (2009), were used for molecular genotyping (Supplementary Table S1). Forward primers were fluorescently 5′-labeled and synthesized by Thermo Fisher Scientific. Reverse primers were synthesized by Eurofins Genomics. SSR loci were amplified in two multiplex PCR reactions using the Type-it microsatellite PCR kit (QIAGEN) in a total volume of 11 μl containing 1.10 μl primer mix (2.0 μm of each primer), 1.10 μl Q-solution, 5.50 μl Type-it Multiplex PCR Master Mix, 2.30 μl Nuclease-Free H_2_O and 1 μl of undiluted DNA. The PCR was run at 95°C for 5 min followed by 12 cycles at 95°C for 30 s, 63°C (−0.5°C per cycle) for 90 s and 72°C for 30 s, followed by 23 cycles at 95°C for 30 s, 57°C for 90 s and 72°C for 30 s and a final extension step at 72°C for 10 min. The PCR program was run on Applied Biosystems™2,720 Thermal Cycler (Thermo Fisher Scientific). PCR reactions were diluted 1:200 in Nuclease-Free H_2_O before size fractionation on Applied Biosystems^™^ 3,730 DNA analyzer (Thermo Fisher Scientific) using the service at *KIGene*, Karolinska University Hospital, Stockholm, Sweden. The amplicons were visualized in GeneMarker^®^ (Softgenetics), and allele sizes (Supplementary Tables S1, S2) were manually scored using the GeneScan^™^ 600 Liz ^®^ Size Standard (Thermo Fisher Scientific).

### Race Identification

A subset of live bulk samples were selected according to year, country, location, host origin and SSR genotype, which resulted in 256 representative isolates that were successfully race phenotyped using a core set of 20 North American stem rust differential lines according to Jin et al. (2008). Four seedlings of each differential line were sown in pots with a 1:1 Pindstrup Substrate peat mix containing slow-release plant nutrients (Pindstrup Mosebrug A/S, Ryomgaard Denmark). The plants were grown in spore-proof cabins in the greenhouse at 17°C during day/12°C during night until full development of first leaf and second leaf half unfolded (approx. 11 days after sowing). Spore samples from cold storage were heat-shocked at 43°C for 5 min. Inoculation of the seedlings was done by spraying 4–5 mg of suspended urediniospores in 5 ml engineered fluid 3 M^™^ Novec^™^ 7,100, (3 M, St. Paul, MN, United States) using an airbrush spray gun (standard class, Revell GmbH, Bünde, Germany) in a biosafety cabinet, followed by mist water to ensure dew formation. Seedlings were incubated in a dew chamber at 18°C in darkness for 24 h, then transferred to spore-proof cabins in the quarantine greenhouse with a 16 h photoperiod of natural light and supplemental sodium light (200 μmol s^−1^ m^−2^), temperature of 20–22°C day/18–20°C night and RH of 80–90%.

Seedling infection types (IT) were scored on the first and second leaf 17 days post-inoculation using a 0–4 scale (Stakman et al., 1962; McIntosh et al., 1995). Isolates conferring ‘low’ ITs, i.e., 0, 0; 1, 1+, 2, and 2+, or combinations thereof, were considered ‘avirulent’ (incompatible), whereas ITs of 3-, 3, 3+, and 4 were considered ‘high’ (i.e., compatible, ‘virulent’; Supplementary Table S3). In case of distinct different ITs on individual differential lines, which could indicate the presence of more than a single race, one to three single pustules were re-isolated. This procedure was repeated until no further signs of mixed ITs were observed in subsequent race assays. Subsets of isolates representing prevalent clades and races, and four unique races from Spain were tested on 35 additional lines representing diverse sources of stem rust resistance (Supplementary Table S4). The seeds were supplied by Cereal Disease Laboratory, USDA, and Agriculture and Agri-Food Canada (AAFC) and multiplied at GRRC; sporadic morphological off-type plants were discarded prior to subsequent seed multiplication. The race typing was generally repeated two to three times, and race nomenclature was based on a modified letter code (Roelfs and Martens, 1984) proposed by Jin et al. (2008). Morocco and McNair were used as susceptible controls in all experiments.

### Impact of Races on Wheat Susceptibility

A set of 53–55 European wheat varieties from 18 breeding companies in 12 countries were evaluated for reaction to seven *Pgt* races representing different genetic groups: Clade III-B (TTRTF, IT76c-18), Clade IV-B (TKTTF, ES34-20), Clade IV-F (TKKTF, DK221a-19), Clade I (TTKSK, KE305c-17 and TTKTT, KE181a-18), Clade VIII (RFCNC, CZ59-20), and an unnamed clade (SKGBR, ES178b2–19). Six seedlings per accession were inoculated under the experimental conditions and procedures described above for race identification. Infection types were scored on the first and second leaf 17 days post-inoculation using the 0–4 scale, where host lines with IT responses below 2+ were considered ‘resistant,’ ITs 2+ and 3- were considered ‘intermediate’ and lines with ITs of 3 and above were considered ‘susceptible.’ Wheat cultivars Morocco and McNair were used as susceptible control (Supplementary Table S5).

### Compilation of Dataset

Duplicate single pustule isolates derived from a bulk sample were removed from the dataset, except when the results revealed multiple genotypes or races within the original sample. Samples that failed recovery were genotyped in case of none or very few samples were recovered from a certain location and year.

### Population Genetic Analyses

Simple Sequence Repeat marker results were investigated through a series of population genetic analyses. The reliability of SSR markers describing the population structure was assessed through the detection of multilocus genotypes (MLGs) plotted against the number of loci using the “*poppr*” package version 2.9.3 implemented in the R environment (Kamvar et al., 2014, 2015). Expected and observed heterozygosity, standard error (SE) of these and inbreeding coefficients (F_IS_ = (Mean H_e_ - Mean H_o_) /Mean H_e_) were calculated in GenAlEx 6.5 (Peakall and Smouse, 2012). The genetic diversity within genetic groups as well as within populations was assessed by the number of MLGs detected within each group using the “*poppr*” package version 2.9.3 implemented in the R environment (Kamvar et al., 2014, 2015). The Shannon–Wiener index (H) was used to measure genotypic diversity (Shannon, 2001). The standardized index of association (rbarD) among SSR loci with 1,000 permutations was calculated to test for association among SSR loci and infer the mode of reproduction of the different populations (Agapow and Burt, 2001). To avoid the effect of epidemic clonality, calculations were repeated using clone-corrected datasets, in which only one representative of each repeated MLG was considered.

The population structure of isolates of different geographical origin was investigated by Discriminant Analysis of Principal Components (DAPC), a multivariate method implemented in the “*adegenet”* R package version 2.1.5 (Jombart, 2008; Jombart et al., 2010). The *find.clusters* function was used to determine the *de novo* optimal number of clusters (*K*), which is normally associated with the lowest value in the ‘elbow’ of the Bayesian Information Criterion (BIC) curve. A cross-validation function (*xvalDapc*) with 1,000 replicates, associated with the lowest root mean square error, was used to define the optimal number of principal components (PCs) to be retained in the DAPC analysis. Membership probabilities of individual isolates associated with a specific MLG and genetic cluster were calculated using the “*adegenet”* R package. The relationship of isolates of different MLGs, including reference isolates representing previously defined genetic groups (Table 2), was calculated using Bruvo distance and visualized in an unrooted Neighbor Joining tree using 2000 bootstraps in the “*poppr*” R package using a cut-off value of 75%.

## Results

**Figure 1:**
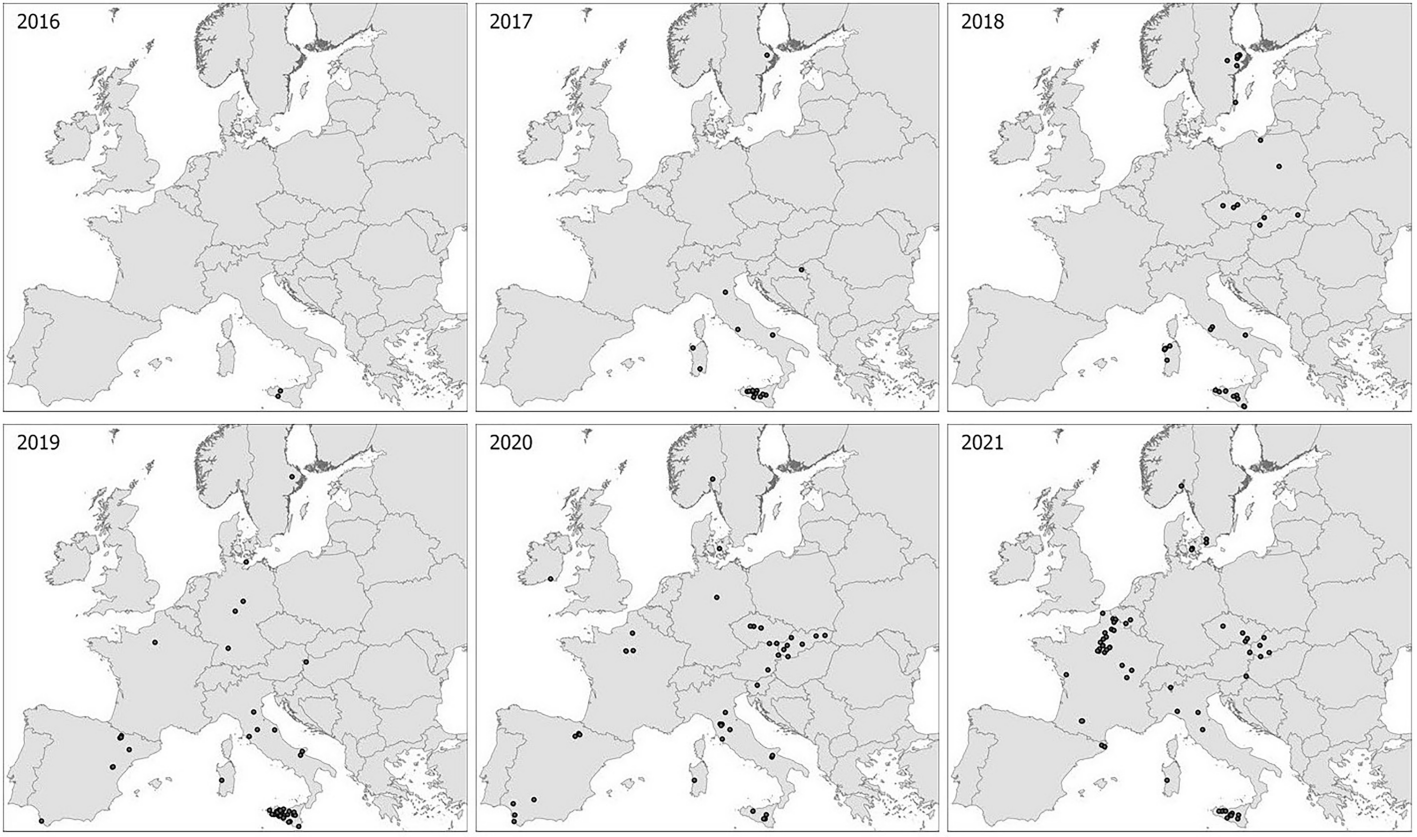
Sampling sites in Europe 2016–2021. Each dot represents a sampling site where one or more samples were collected for genotype/race analyses. Additional sampling sites near Omsk and Novosibirsk in Western Siberia not shown.

The spatial and temporal distribution of samples of wheat stem rust in this study reflected the large differences in disease prevalence across countries and years, but with a clear trend of increasing incidence of stem rust on wheat in Europe since 2016 (Figure 1; Table 1). Relatively high sampling intensity was made during epidemic situations in southern Italy and western Siberia (Russia), and from non-epidemic situations in most other areas with few and sporadic local outbreaks. Samples from Spain represented two contrasting ecological zones, i.e., areas with nearby plants of alternate hosts of *Berberis* spp., and other areas distant from such plants. In this study, stem rust was not detected in France until 2019 (one sample from a single location), which increased to 40 samples from 28 locations in 2021. Eleven isolates from the outbreak in Germany (2013), Denmark (2013) and Sweden (2014) and six isolates from a local study in Sweden by Berlin et al. (unpublished data) were included as references to earlier and ongoing national studies.

More than 80% of the samples considered were successfully recovered (*cf.* Table 1), which provided a unique opportunity to connect information about genetic variability in SSR genotype and virulence phenotype. A total of 540 isolates (2016–2021) were successfully genotyped, of which 81 represented duplicate isolates of identical genotypes/races derived from individual bulk samples. Additionally, 46 samples showed signs of more than a single genotype per sample, mainly from areas with high pathogen diversity. None of the 81 duplicate isolates were considered further, leaving a total of 459 isolates for onwards analyses. Three genotypes detected in the outbreaks of stem rust in Europe 2013–2014 were resampled, i.e., Clade IV-E.1, Clade IV-E.2 and Clade VIII.

**Figure 2:**
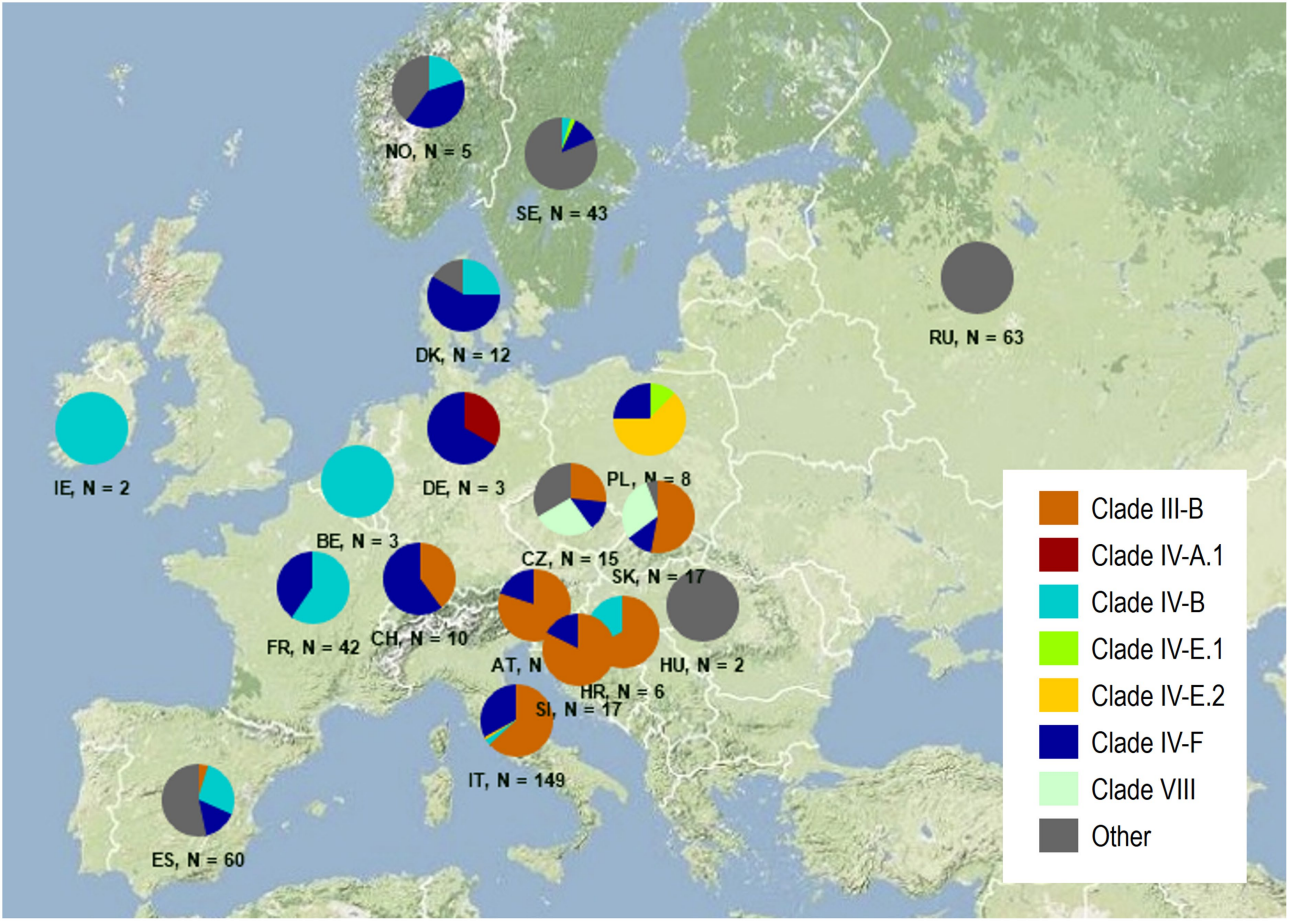
Frequency distribution of genetic groups among 459 isolates of *Puccinia graminis* f. sp. *tritici* in Europe between 2016 and 2021. The designation of distinct groups follows the nomenclature suggested by Szabo et al. (2022). ‘Others’ refer to genetic groups, which have not yet been named internationally. Downloaded 3 February 2022.

The majority of the isolates were assigned to four distinct and previously defined clades, which were often present in multiple countries (Figure 2). Isolates of Clade III-B were the most common, containing one prevalent and four additional MLGs, but only a single race known as the ‘Sicily race,’ TTRTF (Table 3). In this study, TTRTF was first detected in Sicily (2016), but in 2017 and later years, the race was also detected in mainland Italy and Sardinia, Austria, Croatia, Czech Republic, Slovak Republic, Slovenia, and Spain. Race TTRTF has virulence to *Sr13b*, of particular relevance for durum wheat, *Sr35*, *Sr37* and *Sr50* (Supplementary Table S4). Clade IV-F, consisting of a single MLG and race (TKKTF), was first detected in 2018 in this study (Italy, Poland and Sweden). Clade IV-F has spread to a total of 13 European countries by 2021, but so far not associated with epidemics outside Italy. Clade IV-B containing race TKTTF was first detected in 2017 in Europe (Croatia), and in subsequent years in seven additional countries, including Ireland (2020), where stem rust is rarely observed. Clade I (Ug99 race group) was not detected in this study. The results confirm the spreading of relatively few clones across multiple countries in northern and western Europe in recent years, giving rise to sporadic incidences of stem rust on wheat in some countries, and more systematic and widespread occurrence in countries like Italy and France (2021).

**Table 3:** Races detected in Europe within previously defined genetic groups (clades) and number of MLGs in these.

Clade name	Number of MLGs	Race name	Virulence profile	Total
Clade III-B	5	TTRTF	5,21,9e,7b,11,6,8a,9g,36,9b,-,17,9a,9d,10,Tmp,-,-,38,McN	56
		n.a.		79
Clade IV-A.1	1	TKTTF	5,21,9e,7b,-,6,8a,9g,36,9b,30,17,9a,9d,10,Tmp,-,-,38,McN	1
Clade IV-B	1	TKTTF	5,21,9e,7b,-,6,8a,9g,36,9b,30,17,9a,9d,10,Tmp,-,-,38,McN	24
		TKRTF	5,21,9e,7b,-,6,8a,9g,36,9b,-,17,9a,9d,10,Tmp,-,-,38,McN	1
		n.a.		30
Clade IV-E.1	1	TKKTF	5,21,9e,7b,-,6,8a,9g,-,9b,30,17,9a,9d,10,Tmp,-,-,38,McN	2
Clade IV-E.2	1	TKKTF	5,21,9e,7b,-,6,8a,9g,-,9b,30,17,9a,9d,10,Tmp,-,-,38,McN	6
		n.a.		1
Clade IV-F	1	TKKTF	5,21,9e,7b,-,6,8a,9g,-,9b,30,17,9a,9d,10,Tmp,-,-,38,McN	36
		n.a.		69
Clade VIII	2	RFCNC	5,21,-,7b,-,-,8a,9g,-,-,-,17,9a,-,10,-,-,-,-,McN	11
		RFCPC	5,21,-,7a,-,-,8a,9g,-,-,-,17,9a,-,10,Tmp,-,-,-,McN	2
		n.a.		5
Other	92	= 76 races		108
		n.a.		28
Total	104			459

*‘Other’ represents 76 races among 92 MLGs detected in the genetic group; n.a.: not race typed*.

Multiple genetic groups, which have not been named previously, were termed ‘Others’ (Figure 2; Table 3). These groups were prevalent in local areas in Sweden, Spain, and Russia, often sampled from cereals in proximity to the alternate host, *Berberis* spp. Many of the races were characterized by avirulence on multiple wheat differential lines (Supplementary Tables S3, S4) and some of the isolates revealed unexpected, intermediate infection types. In summary, the results suggested the presence of local highly diverse *Pgt* populations in Russia, Spain and Sweden consisting of multiple races, and in most other areas, clonal populations consisting of only few genetic groups and races.

### Genetic Diversity

The genetic structure of *Pgt* was investigated for seven geographically and ecologically different sampling areas (populations), i.e., Italy, Spain (Barberry area), Spain (non-Barberry area), Sweden, Russia (Siberia), France and Europe (other). The latter represents 13 countries (*cf.* Table 1), where wheat stem rust was observed only sporadically, resulting in relatively few samples compared to a large geographical sampling area (Table 4).

**Table 4:** Genetic diversity of geographical spaced populations of *Puccinia graminis* f.sp. *tritici* in Europe and Russia.

Geographical population	Number of samples (***n***)	Number of MLGs	Number of MLG/n	Shannon–Weiner H index	rbarD	
					Value	Value of *p*
Italy	150	7	0.047	0.944	0.469	0.001
France	42	2	0.048	0.675	0.983	0.001
Spain non-barberry	26	4	0.154	1.003	0.515	0.001
Spain barberry	32	28	0.875	3.292	0.012	0.044
Sweden	43	25	0.581	2.943	0.087	0.001
Russia	63	40	0.635	3.160	<0.001	0.471
Other (Europe)	103	11	0.075	1.672	0.104	0.002

*rbarD based on clone-corrected data*.

**Figure 3:**
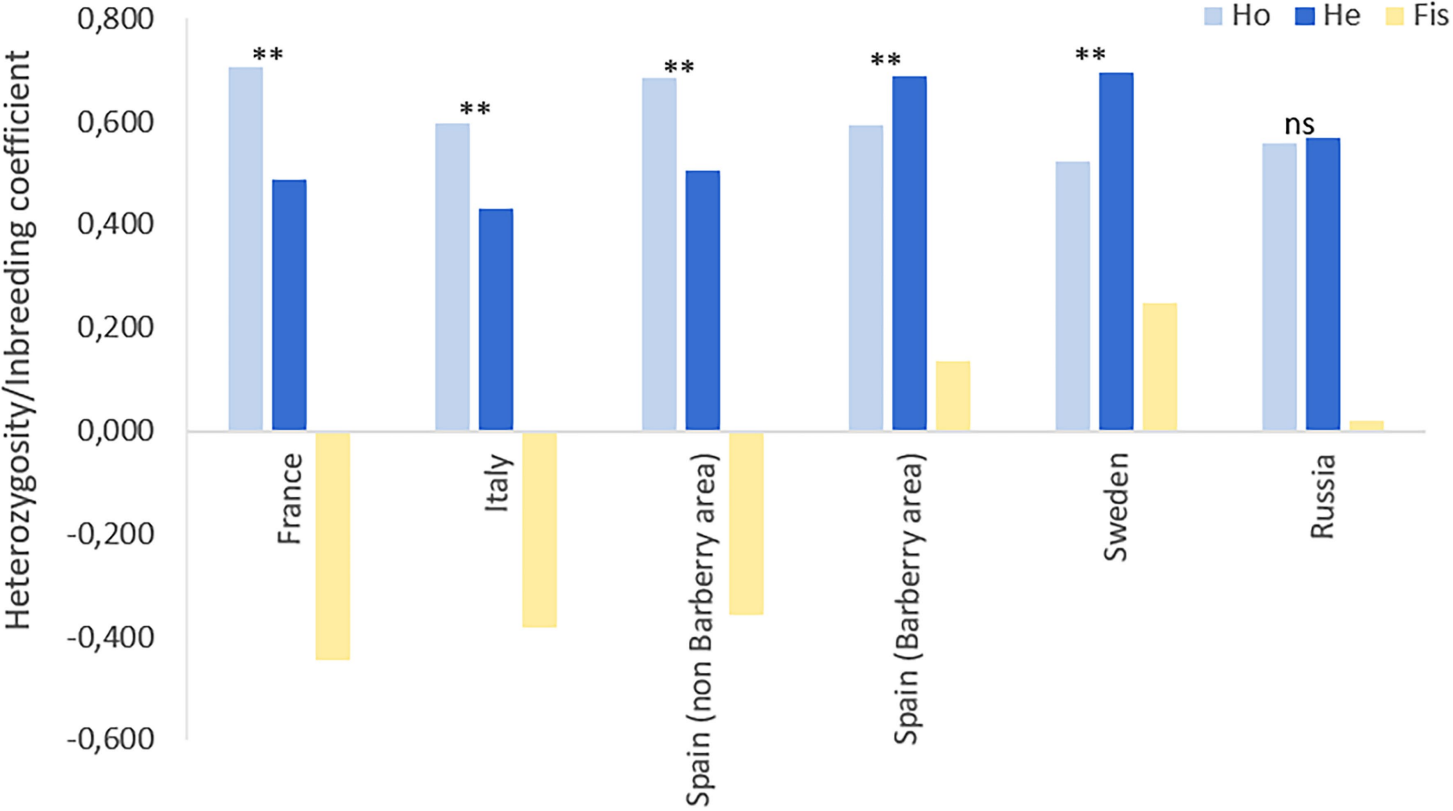
Observed heterozygosity (H_o_), expected heterozygosity (H_e_) and inbreeding coefficients (F_is_) of geographical spaced populations of *Puccinia graminis* f.sp*. tritici* in Europe and Siberia. Samples from outside these areas were not considered due to low sample sizes in individual countries. Level of significance: *p* < 0.01^**^.

The 17 SSR primers revealed a total of 104 multilocus genotypes (MLGs) based on a total of 179 different alleles (Table 4; Supplementary Figure S1). The seven populations revealed very different degree of genetic diversity. The populations in Italy, Spain (non-barberry area) and France had low number of genotypes compared to sampling size, low Shannon–Weiner diversity index, high level of linkage disequilibrium (Table 4) and excess of observed heterozygosity as compared to the expected under Hardy–Weinberg (HW) equilibrium (Figure 3). These features are strong signatures of clonal reproduction. In contrast, the populations from Spain (barberry area), Sweden and Russia showed strong signatures of sexual reproduction, revealed by high numbers of genotypes compared to sampling size, high diversity indexes and linkage disequilibria close to zero in Russia and slightly higher in Spain (barberry area) and Sweden. Observed/expected heterozygosity were in HW proportions for Russia or lower than expected for Spain (barberry) and Sweden, which may indicate some degree of inbreeding in the last two mentioned. This is in line with F_IS_ values of 0.14 and 0.25, respectively, for these two populations as opposed to 0.019 for Russia and negative values for the clonal populations in Italy, France and Spain (non-barberry; Figure 3). Samples from other European countries, representing relatively few samples collected across several years, had parameter values between these extremes, which suggests that regions in Europe, where stem rust is not yet established, may be influenced by incursions of individuals of diverse evolutionary origin.

**Figure 4:**
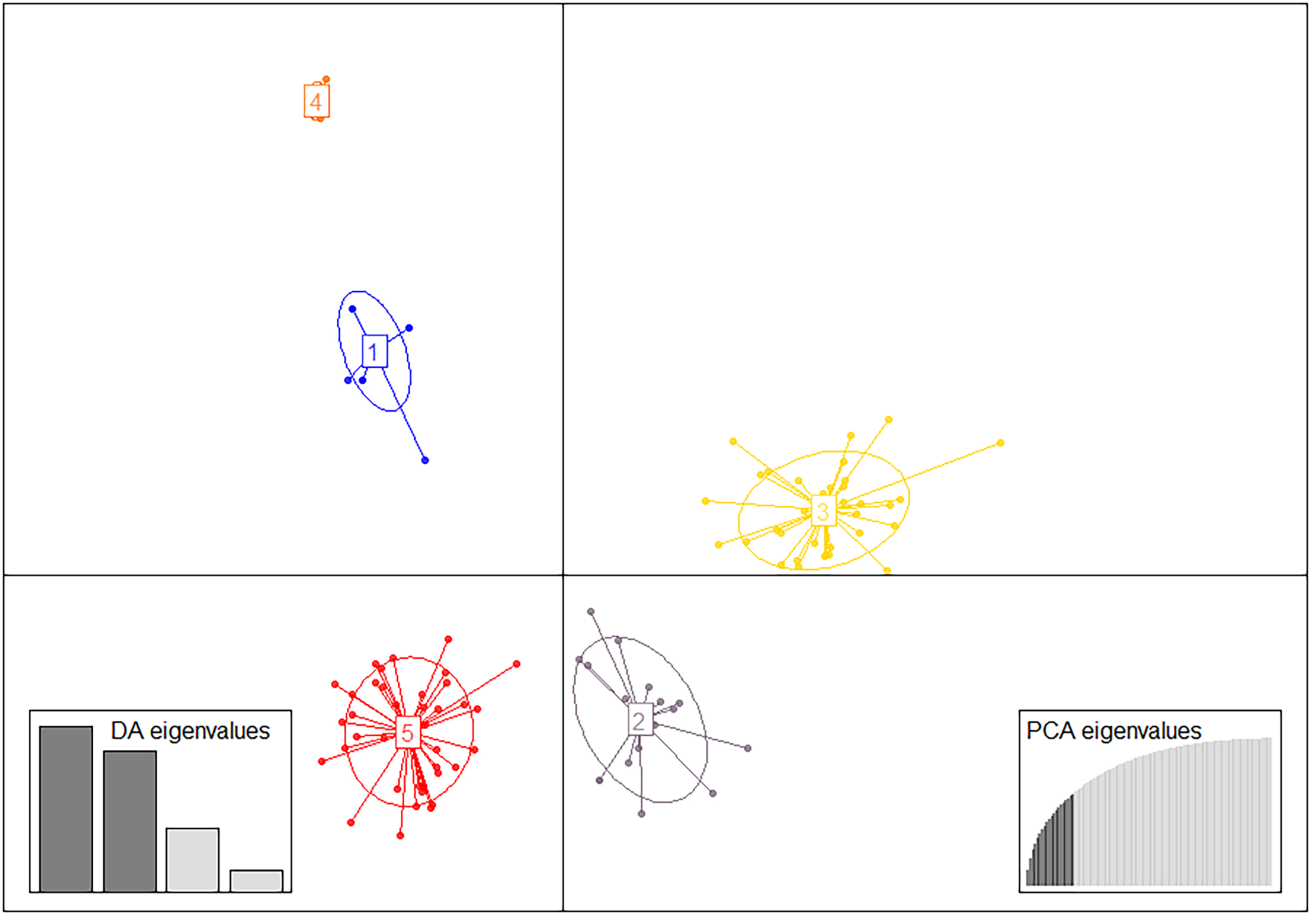
Discriminant analysis of principal components (DAPC) for the 104 multilocus genotypes (MLGs) detected (clone corrected data). The axes represent the first two linear discriminants. Each ellipsis represents distinct genetic clusters, and dots represent individuals associated with unique MLGs. Eigenvalues indicate the amount of genetic information retained by the PCA (left inset) and the discriminant function (DA, right inset).

The DAPC analysis revealed five genetic clusters based on 20 retained PCs and six discriminant (DA) eigenvalues, which were sufficient to capture the genetic structure of the whole dataset (Figure 4; Supplementary Figure S2). This corresponded with the optimal value of BIC indicated by the lowest value of the elbow observed in the BIC curve (Supplementary Figure S3). The 104 MLGs detected among the 459 isolates included in the study were clustered in the DAPC analyses based on membership probabilities larger than 0.9999 (Supplementary Table S6). Cluster 1 consisted of previously defined clades, such as Clade IV-A.1, Clade IV-B, Clade IV-F, Clade IV-E.1, and Clade IV-E.2. Cluster 4 consisted of five closely related MLGs in Clade III-B. Both clusters (CL1 and CL4), consisting of previously defined groups, were clearly separated from the three others. Cluster 5 consisted of a diverse population from Russia (western Siberia) and Clade VIII consisting of only two MLGs mainly detected in eastern Europe. Cluster 2 consisted of MLGs representing isolates from Sweden, a single MLG detected in Norway, a different MLG detected in Czech Republic and a re-sampled MLG detected in Denmark. Finally, Cluster 3 consisted of 28 MLGs from Spain and 8 MLGs from Sweden.

**Figure 5:**
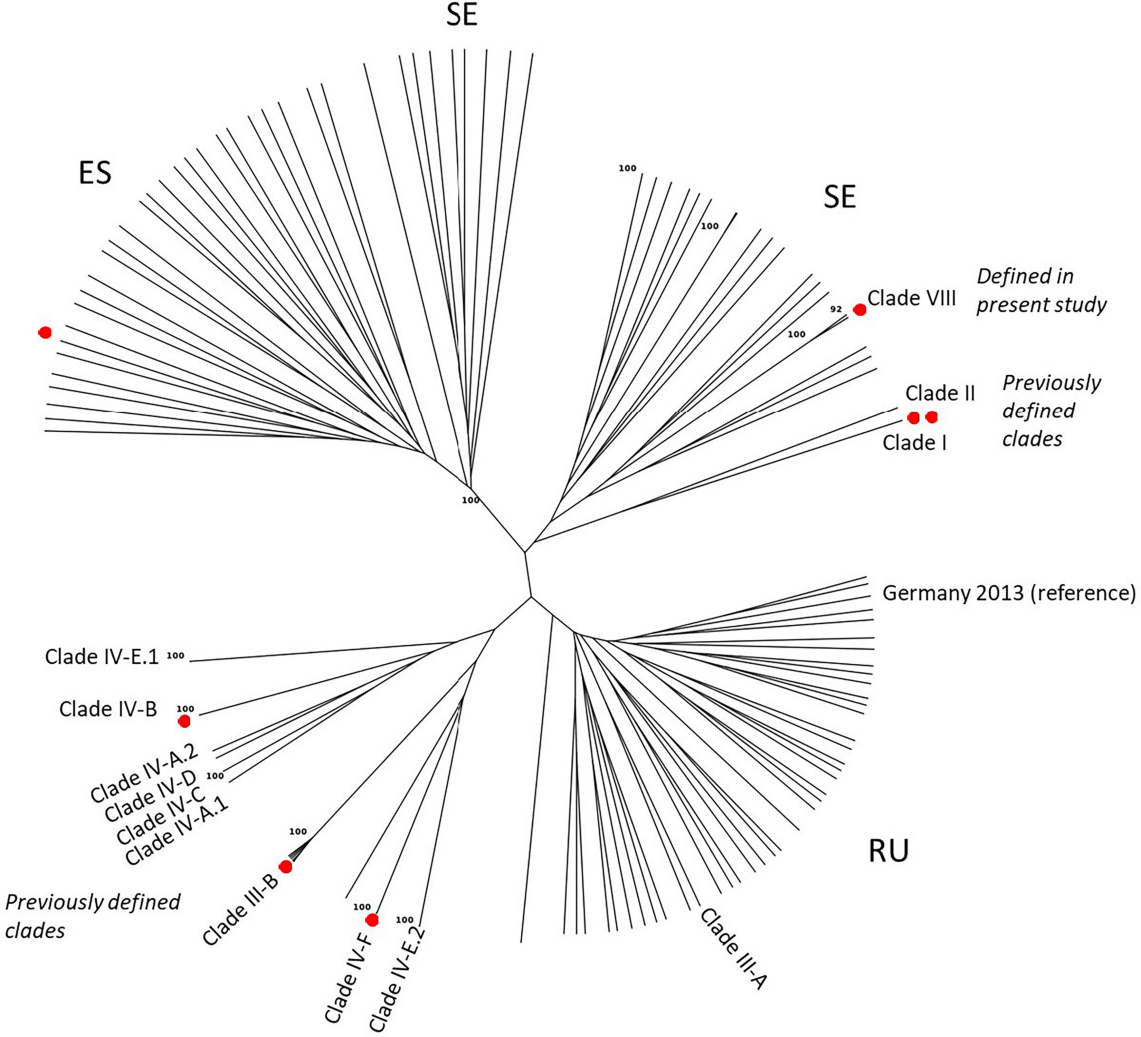
Unrooted Neighbor Joining tree (clone-corrected data) using Bruvo’s distance showing the genetic relationship of European stem rust isolates (2016–2021) and additional reference isolates representing previously defined genetic groups (Clades) detected in Europe and elsewhere. National letter codes are indicative: ES (Spain), SE (Sweden), RU (Russia). Red dots represent isolates used for assessment of host susceptibility.

The overall conclusions from the DAPC analyses were confirmed by phylogenetic analyses (Figure 5), which also comprised reference isolates representing genetic groups reported in previous European and international studies (*cf.* Table 2). One branch consisted of already defined clades, most of which have been previously detected in Europe, i.e., clade IV-A.1, IV-A.2, IV-E.1, IV-E.2, and IV-F (equivalent to CL1, Figure 4). Clade III-B formed a unique group of five closely related MLGs, equivalent to CL4 in Figure 4. A second branch consisted of 38 diverse MLGs from two local areas near Omsk and Novosibirsk in western Siberia, Russia, which corresponded with CL5 in Figure 4. A Clade III-A reference isolate and an isolate from the German outbreak in 2013 (unpublished) were part of CL5. Whereas the two MLGs in the newly defined Clade VIII grouped with the Russian isolates in the DAPC analysis, this was not the case in the NJ tree, where they were located within a third branch consisting of genetically diverse isolates from Scandinavia and Czech Republic (CL2 in Figure 4). A fourth branch consisted of 36 MLGs representing isolates from Spain and Sweden, corresponding to CL3 (Figure 4).

In conclusion, the local sexual populations in Spain (barberry area), Sweden and Russia, respectively, were highly diverse and formed distinct genetic groups. These were clearly separated from previously named and prevalent clades, each consisting of one or few races and/or MLGs. One MLG from Clade VIII, which was already detected in the German outbreak in 2013, was resampled in Czech Republic, Slovakia and Hungary in the present study, and in another case, an MLG first detected in a sexual population in Sweden (2019) was detected in Denmark (2020). Other reference isolates such as Clade I (Ug99), Clade II, Clade III-A, Clade IV-C and Clade IV-D, were not detected in this study.

### Diversity by Virulence in Local Populations in Spain, Sweden, and Russia

In addition to typical races found in Europe, samples from barberry areas in Spain, Sweden, and Russia revealed a high number of unique races in low frequencies and unusual virulence combinations, and occasional unexpected infection types (IT) on specific combinations of differential lines (Supplementary Tables S3, S4).

In Spain, 54 of 58 genotyped isolates were successfully virulence phenotyped based on the IT responses on 20 wheat lines collectively assembled to differentiate races in *Pgt* (Table 5). The results were highly dependent on sampling area, i.e., high race diversity in areas in proximity of alternate hosts of *Berberis* spp. (28 races among 30 isolates), and low diversity in areas where the alternate host was not detected near sampling sites (4 races among 24 isolates). In general, different races were represented by different genotypes (MLGs). Only in four cases, different races shared the same genotype, i.e., races within MLG ID number 6, 11, 23 and 89, respectively (Table 5).

**Table 5:** Virulence diversity of 31 races of *Puccinia graminis* f.sp. *tritici* in areas in Spain in proximity of *Berberis* species and in areas where the alternate host was absent.

Area	Race profile no.	Host	Virulence formula	Race name	SSR name	MLG ID	Total
Barberry area	1	Bread wheat	-,21,9e,-,11,6,8a,9 g,-,9b,-,17,-,-,-,-,-,31,38,McN	JTHBK	Other	92	1
	2	Elymus repens	-,21,9e,7b,-,6,8a,9 g,-,9b,-,-,-,-,-,-,-,31,38,McN	KKGBK	Other	62	1
	3	Bread wheat	-,21,9e,7b,-,6,8a,9 g,-,9b,-,-,-,-,-,-,24,-,-,McN	KKGBM	Other	12	1
	4	Bread wheat	-,21,9e,7b,-,6,8a,9 g,-,9b,-,-,9a,-,-,-,24,-,38,McN	KKGLP	Other	35	1
	5	Bread wheat	5,-,9e,7b,-,6,8a,9 g,-,9b,-,-,9a,-,-,-,-,31,-,McN	PKGLH	Other	39	1
	6	Rye	5,-,9e,7b,11,6,8a,9 g,-,9b,-,-,-,-,-,-,-,31,-,McN	PTGBH	Other	11	1
	7	Rye	5,-,9e,7b,11,6,8a,9 g,-,9b,-,-,-,-,10,Tmp,-,31,-,McN	PTGFH	Other	11	1
	8	Rye	5,21,-,7b,-,6,8a,9 g,-,-,-,-,-,-,-,-,-,31,38,McN	RKBBK	Other	16	1
	9	Bread wheat	5,21,9e,-,-,-,-,9 g,-,9b,-,-,-,-,-,-,-,-,-,-	SCGBB	Other	9	1
	10	Bread wheat	5,21,9e,-,-,6,8a,9 g,-,-,-,-,-,-,-,-,24,-,38,McN	SKBBP	Other	13	1
	11	Rye	5,21,9e,-,-,6,8a,9 g,-,9b,-,-,-,-,-,-,-,-,-,McN	SKGBC	Other	45	1
	12	Elymus repens	5,21,9e,-,-,6,8a,9 g,-,9b,-,-,-,-,-,-,-,-,38,McN	SKGBF	Other	4	1
	13	Rye	5,21,9e,-,-,6,8a,9 g,-,9b,-,-,-,-,-,-,-,31,-,McN	SKGBH	Other	22	1
	14	Bread wheat	5,21,9e,-,-,6,8a,9 g,-,9b,-,-,-,-,-,-,24,31,-,McN	SKGBR	Other	41	1
	15	Rye	5,21,9e,-,-,6,8a,9 g,-,9b,-,-,-,-,-,-,24,31,-,McN	SKGBR	Other	89	1
	16	Elymus repens	5,21,9e,-,-,6,8a,9 g,-,9b,-,-,-,-,-,-,24,31,38,McN	SKGBT	Other	15	1
					Other	19	1
	17	Elymus repens	5,21,9e,-,11,6,-,9 g,-,9b,-,-,9a,-,-,-,-,31,-,McN	SRGLH	Other	7	1
	18	Rye	5,21,9e,7b,-,6,8a,9 g,-,-,30,-,9a,-,-,-,-,31,38,McN	TKDLK	Other	6	1
	19	Bread wheat	5,21,9e,7b,-,6,8a,9 g,-,9b,-,-,-,-,-,-,-,31,-,McN	TKGBH	Other	2	1
	20	Rye	5,21,9e,7b,-,6,8a,9 g,-,9b,-,-,-,-,-,-,-,31,38,McN	TKGBK	Other	5	1
	21	Rye	5,21,9e,7b,-,6,8a,9 g,-,9b,-,-,-,-,-,-,24,31,-,McN	TKGBR	Other	89	1
	22	Rye	5,21,9e,7b,-,6,8a,9 g,-,9b,-,-,-,-,10,-,-,31,-,McN	TKGDH	Other	6	1
	23	Bread wheat	5,21,9e,7b,-,6,8a,9 g,-,9b,-,-,9a,-,-,-,-,31,-,McN	TKGLH	Other	46	1
	24	Bread wheat	5,21,9e,7b,-,6,8a,9 g,-,9b,-,-,9a,-,-,-,-,31,38,McN	TKGLK	Other	10	1
	25	Bread wheat	5,21,9e,7b,-,6,8a,9 g,-,9b,-,17,-,-,10,-,24,31,-,McN	TKHDR	Other	3	1
					Other	93	1
	26	Bread wheat	5,21,9e,7b,11,-,8a,9 g,-,-,-,-,9a,-,-,-,-,31,-,McN	TPBLH	Other	23	1
	27	Bread wheat	5,21,9e,7b,11,6,8a,9 g,-,9b,-,-,9a,-,-,-,-,31,-,-	TTGLG	Other	23	1
	28	Bread wheat	5,21,9e,7b,11,6,8a,9 g,-,9b,-,17,9a,-,-,-,-,31,-,McN	TTHLH	Other	99	1
Non-Barberry area	29	Bread wheat	5,21,9e,7b,-,6,8a,9 g,-,9b,30,17,9a,9d,10,Tmp,-,-,38,McN	TKKTF	Clade IV-F	103	2
		Durum wheat	5,21,9e,7b,-,6,8a,9 g,-,9b,30,17,9a,9d,10,Tmp,-,-,38,McN				4
	30	Bread wheat	5,21,9e,7b,-,6,8a,9 g,36,9b,-,17,9a,9d,10,Tmp,-,-,38,McN	TKRTF	Clade IV-B	101	1
	31	Bread wheat	5,21,9e,7b,-,6,8a,9 g,36,9b,30,17,9a,9d,10,Tmp,-,-,38,McN	TKTTF	Clade IV-B	101	12
		Durum wheat	5,21,9e,7b,-,6,8a,9 g,36,9b,30,17,9a,9d,10,Tmp,-,-,38,McN				1
		Spelt	5,21,9e,7b,-,6,8a,9 g,36,9b,30,17,9a,9d,10,Tmp,-,-,38,McN				1
	32	Bread wheat	5,21,9e,7b,11,6,8a,9 g,36,9b,-,17,9a,9d,10,Tmp,-,-,38,McN	TTRTF	Clade III-B	28	1
		Durum wheat	5,21,9e,7b,11,6,8a,9 g,36,9b,-,17,9a,9d,10,Tmp,-,-,38,McN				2
Total							54

*Shaded cells represent Sr31-virulent races*.

The presence of *Sr31*-virulence in 22 races was striking, irrespective of host origin, representing wheat, rye, and a wild grass (*Elymus repens*). A subset of four of these isolates were tested on 35 additional wheat lines representing diverse and occasionally newly identified sources of resistance (Supplementary Table S4). These results confirmed highly unusual and different virulence patterns, including confirmation of virulence to *Sr31* using three wheat lines, Federation^*^4/Kavkaz, Sr31/6^*^LMPG and Siouxland, as well as virulence to *Sr22, Sr24, Sr44*, *Sr45*, *Sr49*, and *Sr50*. In addition, we observed higher than expected ITs for three incompatible interactions involving *Sr31* (2, 2+ and 22+), *Sr24* (22+), and *Sr50* (2-). The fact that *Sr31*-virulent races were observed for samples collected from three host genera (*Triticum*, *Secale* and *Elymus*), and the lack of differentiation according to host origin with respect to virulence/race pattern in general, demonstrated that wheat adapted stem rust from local areas in Spain were able to infect and reproduce on rye and wild grasses.

In contrast, the samples from agricultural areas separated from alternate hosts, *Berberis* spp., revealed only four races; TKTTF (from bread wheat, durum wheat and spelt), TKRTF (bread wheat), and TKKTF and TTRTF, the latter two collected from both bread wheat and durum wheat. The four races were assigned to three clades that were also detected at multiple locations outside Spain (Figure 2).

Twenty-nine live isolates from Sweden revealed 13 race phenotypes, representing five reference samples from a local study in Sweden (2017), two collections from barley in 2018 and one collection from wheat in 2019 and 2020 (Table 6). A single race/genotype (QCHNC; MLG40) was resampled twice from a single wheat plot in Denmark (2020).

**Table 6:** Virulence diversity of 13 races of *P. graminis* f. sp. *tritici* in Sweden 2017–2021.

Race profile no.	Host	Virulence formula	Race name	SSR name	MLG ID	Total
1	Bread wheat	5,-,-,-,-,-,8a,9 g,-,-,-,17,9a,-,-,-,-,-,-,McN	LFCLC	Other	98	1
2	Barley	5,-,-,-,-,-,8a,9 g,-,-,-,17,9a,-,10,-,-,-,-,McN	LFCNC	Other	8	1
	Bread wheat			Other	**83**	1
3	Barley	5,-,-,-,-,-,8a,9 g,36,-,-,17,9a,-,10,-,-,-,-,McN	LFMNC	Other	68	3
4	Barley	5,-,-,7b,-,-,8a,9 g,-,-,-,17,9a,-,10,-,-,-,-,McN	MFCNC	Other	**61**	1
	Bread wheat				1	1
	Bread wheat				14	1
5	Barley	5,-,-,7b,-,-,8a,9 g,36,-,-,17,9a,-,10,Tmp,-,-,-,McN	MFMPC	Other	68	2
6	Bread wheat	5,21,-,-,-,-,-,9 g,-,9b,-,17,9a,-,10,-,-,-,-,McN	QCHNC	Other	40	1
7	Barley	5,21,-,-,-,6,8a,9 g,-,9b,-,17,9a,-,-,-,-,-,-,McN	QKHLC	Other	90	3
	Barley			Other	91	2
8	Barley	5,21,-,-,-,6,8a,9 g,36,9b,-,17,9a,-,-,-,-,-,-,McN	QKRLC	Other	90	3
9	Bread wheat	5,21,-,7b,-,-,-,9 g,-,-,-,17,9a,-,10,Tmp,-,-,-,McN	RCCPC	Other	95	4
10	Bread wheat	5,21,-,7b,-,-,8a,9 g,-,-,-,17,9a,-,10,Tmp,-,-,-,McN	RFCPC	Other	**37**	1
11	Bread wheat	5,21,-,7b,-,-,8a,9 g,-,-,-,17,9a,9d,10,Tmp,-,-,-,McN	RFCTC	Other	36	1
12	Bread wheat	5,21,9e,7b,-,6,8a,9 g,-,9b,30,17,9a,9d,10,Tmp,-,-,38,McN	TKKTF	Clade IV-E.1	25	1
	Bread wheat			Clade IV-F	103	1
13	Barley	5,21,9e,7b,11,6,8a,9 g,36,9b,-,-,-,9d,10,Tmp,-,-,38,McN	TTQKF	Other	**56**	1
Total						29

*Isolates were derived from samples collected from cultivated barley and bread wheat in areas where Berberis vulgaris was present. Reference samples from Berlin et al. (2021; unpublished) are marked in bold in the MLG ID column*.

TKKTF was detected in two different clades, IV-E.1, and IV-F, but also represented in a reference isolate collected in Sweden in 2014 (Clade IV-E.2). Except for TKKTF and TTQKF, the races in Sweden were characterized by a relatively high number of avirulences. None of the races, except TKKTF and QCHNC mentioned above, were detected outside Sweden. In contrast to Spain, *Sr24-, Sr31*-, and *Sr50*-virulences were not detected. The unusual virulence patterns and complexity of races confirmed the high degree of genotypic diversity and separation from other populations in the study.

Fifty-three live isolates from western Siberia were race phenotyped, representing the majority of samples from two neighboring regions in 2016 and 2017 that were successfully genotyped (Table 7). A total of 35 races were detected, of which only five were found more than once. All races were different from races detected elsewhere in the study, which was confirmed by the fact that none of previously defined clades in Europe, Africa, Asia and North America were detected (Table 7). In general, different races were represented by different genotypes, except MLG 100, which was represented by four races (LCCSF, LCRSF, QCHSF, and QCRSF). Interestingly, a single race has *Sr31*-virulence (race profile no. 8), but is indeed very different from known races of Ug99 and *Sr31*-virulent races from Spain (Table 5, this study). The *Sr31*-virulence was confirmed by repeated tests using *Sr31* resistance in three genetic backgrounds. Overall, the races had different virulence/avirulence pattern ranging from individuals with relatively few virulences, for example race profile 1 (virulences: 5, 9 g, 17, 9a, 9d, 10, 38) to individuals with high number of virulences such as race profile 35 (virulences: 5, 21, 9e, 7b, 6, 8a, 9 g, 36, 9b, 17, 9a, 10, 38, Tmp).

**Table 7:** Virulence diversity of 35 races of *P. graminis* f.sp. *tritici* in Russia 2016–2017.

Race profile no.	Host	Virulence formula	Race name	SSR name	MLG ID	Total
1	Bread wheat	5,-,-,-,-,-,-,9 g,-,-,-,17,9a,9d,10,-,-,-,38,McN	LCCSF	Other	100	3
					50	1
2	Bread wheat	5,-,-,-,-,-,-,9 g,36,9b,-,17,9a,9d,10,-,-,-,38,McN	LCRSF	Other	100	4
3	Durum wheat	5,-,-,-,-,-,8a,9 g,36,-,-,17,9a,9d,10,-,-,-,38,McN	LFMSF	Other	57	1
					55	1
4	Durum wheat	5,-,-,-,-,-,8a,9 g,36,9b,-,17,9a,9d,10,-,-,-,38,McN	LFRSF	Other	60	1
5	Bread wheat	5,-,-,-,-,6,-,9 g,-,-,-,17,9a,9d,10,-,-,-,38,McN	LHCSF	Other	70	1
6	Bread wheat	5,-,-,-,-,6,-,9 g,36,9b,-,17,9a,9d,10,-,-,-,38,McN	LHRSF	Other	66	1
7	Bread wheat	5,-,-,-,-,6,8a,9 g,-,-,-,17,9a,9d,10,-,-,-,38,McN	LKCSF	Other	87	2
					88	1
8	Bread wheat	5,-,-,-,11,6,8a,9 g,-,-,-,-,9a,9d,10,-,-,31,38,McN	LTBSK	Other	21	1
9	Durum wheat	5,-,-,-,11,6,8a,9 g,36,-,-,17,9a,9d,10,-,-,-,38,McN	LTMSF	Other	63	1
10	Bread wheat	5,-,-,7b,-,-,-,9 g,36,-,-,17,9a,9d,10,-,-,-,38,McN	MCMSF	Other	59	1
11	Bread wheat	5,-,-,7b,-,-,8a,9 g,36,-,-,17,9a,9d,10,Tmp,-,-,-,McN	MFMTC	Other	71	1
12	Bread wheat	5,-,-,7b,11,-,8a,9 g,36,-,-,17,9a,9d,10,Tmp,-,-,-,McN	MPMTC	Other	64	1
13	Barley	5,-,9e,-,-,-,8a,9 g,36,-,-,17,9a,9d,10,-,-,-,38,McN	NFMSF	Other	58	1
	Bread wheat					1
14	Bread wheat	5,21,-,-,-,-,-,9 g,-,9b,-,17,9a,9d,10,-,-,-,38,McN	QCHSF	Other	100	4
15	Bread wheat	5,21,-,-,-,-,-,9 g,36,9b,-,17,9a,9d,10,-,-,-,38,McN	QCRSF	Other	100	5
16	Bread wheat	5,21,-,-,-,-,8a,9 g,36,9b,-,17,9a,9d,10,-,-,-,38,McN	QFRSF	Other	42	1
17	Bread wheat	5,21,-,-,-,6,-,9 g,-,9b,-,17,9a,9d,10,-,-,-,-,McN	QHHSC	Other	49	1
18	Bread wheat	5,21,-,-,-,6,-,9 g,-,9b,-,17,9a,9d,10,-,-,-,38,McN	QHHSF	Other	75	1
19	Durum wheat	5,21,-,-,-,6,-,9 g,36,-,-,17,9a,9d,10,-,-,-,38,McN	QHMSF	Other	86	1
20	Bread wheat	5,21,-,-,-,6,8a,9 g,-,-,-,17,9a,9d,10,-,-,-,38,McN	QKCSF	Other	78	1
					79	1
21	Bread wheat	5,21,-,-,-,6,8a,9 g,-,9b,-,17,9a,9d,10,-,-,-,38,McN	QKHSF	Other	44	1
22	Bread wheat	5,21,-,7b,-,-,-,9 g,36,-,-,17,9a,9d,10,Tmp,-,-,38,McN	RCMTF	Other	73	1
23	Bread wheat	5,21,-,7b,-,-,-,9 g,36,9b,-,17,9a,9d,10,Tmp,-,-,38,McN	RCRTF	Other	52	1
24	Bread wheat	5,21,-,7b,-,-,8a,9 g,36,-,30,17,9a,9d,10,Tmp,-,-,38,McN	RFPTF	Other	77	1
25	Bread wheat	5,21,-,7b,-,-,8a,9 g,36,9b,-,17,9a,9d,10,-,-,-,38,McN	RFRSF	Other	24	1
26	Bread wheat	5,21,-,7b,-,6,-,9 g,-,-,-,17,9a,9d,10,Tmp,-,-,38,McN	RHCTF	Other	81	1
27	Bread wheat	5,21,-,7b,-,6,-,9 g,36,9b,-,17,9a,9d,10,Tmp,24,-,38,McN	RHRTP	Other	43	1
28	Bread wheat	5,21,-,7b,-,6,8a,9 g,36,9b,-,17,9a,9d,10,-,24,-,38,McN	RKRSP	Other	47	1
29	Bread wheat	5,21,-,7b,11,6,-,9 g,-,9b,-,-,9a,9d,10,Tmp,-,-,-,McN	RRLTC	Other	97	1
30	Bread wheat	5,21,9e,-,-,-,8a,9 g,36,9b,-,17,9a,9d,10,-,-,-,38,McN	SFRSF	Other	76	1
31	Bread wheat	5,21,9e,-,-,6,-,9 g,-,9b,-,17,9a,9d,10,-,-,-,38,McN	SHHSF	Other	65	1
32	Bread wheat	5,21,9e,7b,-,6,-,9 g,36,9b,-,17,9a,9d,10,Tmp,-,-,-,McN	THRTC	Other	96	1
33	Durum wheat	5,21,9e,7b,-,6,8a,9 g,36,-,-,17,9a,9d,10,Tmp,-,-,38,McN	TKMTF	Other	51	1
34	Bread wheat	5,21,9e,7b,-,6,8a,9 g,36,-,30,17,9a,9d,10,Tmp,-,-,38,McN	TKPTF	Other	26	1
35	Bread wheat	5,21,9e,7b,-,6,8a,9 g,36,9b,-,17,9a,-,10,Tmp,-,-,38,McN	TKRPF	Other	54	1
Total						53

*Isolates were derived from samples collected from cultivated barley, bread wheat and durum wheat near Omsk and Novosibirsk. Shaded cells represent a Sr31-virulent race*.

### Impact of Races on Wheat Susceptibility

Seedling tests using seven isolates representing diverging genetic groups (Figure 5) demonstrated that most of the investigated wheat varieties were susceptible to most of the races (Table 8; Supplementary Table S5). The two Ug99 races revealed susceptibility to the highest number of varieties, whereas the race SKGBR from a recombining population in Spain was less adapted to the varieties investigated. Two durum wheat varieties (Svevo and Marco Aurelio) showed resistance to all races except the Sicily race (TTRTF). Information about the detailed responses of individual varieties to individual races are provided in Supplementary Table S5.

**Table 8:** Seedling response of European wheat varieties to three clades representing prevalent races of *P. graminis* f.sp. *tritici* in Europe, a race from a recombining population in Spain, a race from the German outbreak 2013, and two races representing Ug99 from East Africa.

	Common clades/races (2016–21)			Spain 2019	Germany 2013	Ug99	
	III-B	IV-B	IV-F	unnamed	Clade VIII	Clade I	
Response category^*^	TTRTF	TKTTF	TKKTF	SKGBR	RFCNC	TTKTT	TTKSK
Susceptible	38	40	38	10	15	48	46
Intermediate	8	2	3	10	16	4	0
Resistant	8	11	12	33	21	2	8
Total number	54	53	53	53	52	54	54

* *Varieties with IT below 2+ were categorized ‘resistant,’ IT 2+ and 3- were considered ‘intermediate’ and lines with ITs of 3 and above were ‘susceptible’*.

## Discussion

The present study represents a comprehensive dataset based on SSR genotyping and race phenotyping of more than 400 samples of *Puccinia graminis* f. sp. *tritici*, which were collected in 17 European countries and Siberia between 2016 and 2021. By comparing to results of isolates of non-European origin, hosted by the GRRC,^2^ and reference isolates from the USDA ARS Cereal Disease Lab, we were able to address hypotheses about possible origin and spatial patterns of the re-emergence of wheat stem rust in Europe since the outbreak in Sicily in 2016 (Bhattacharya, 2017). Sampling in local areas in proximity to the alternate host, *Berberis* spp., and in corresponding non-*Berberis* areas, allowed us to investigate the current role of *Berberis* spp. in wheat stem rust epidemiology in Europe. This is a question, which has occupied scientists and plant breeders historically Lind (1915); Stakman (1923); Hermansen (1968) and in recent years the discussion about the potential role of common barberry has been brought up again due to the repeal of *Berberis* eradication laws in several European countries in the 1990s (Berlin et al., 2012) and the replanting of rust susceptible *Berberis* spp. for environmental purposes in the United Kingdom (Saunders et al., 2019). The conclusions in this study were supported by different population genetic and plant pathology approaches, which generally resulted in similar outcomes.

Since the local outbreaks in Germany in 2013 (Olivera Firpo et al., 2017), Sicily in 2016 (Patpour et al., 2020), Sweden in 2017 (Berlin et al., 2012), and Siberia in 2016–2017 (Shamanin et al., 2020), stem rust has been increasingly frequent in northern and western Europe, for example in France (2021), where it has been reported only sporadically since the 1970s (Massenot, 1978). This is consistent with aerial spread of urediniospores across large distances, giving rise to observations of stem rust on wheat in new areas in Europe. The detection of relatively few clonal lineages (genotypes) of particular races, some of which have been detected outside Europe in previous years Olivera et al. (2019),^3^ underline the capacity of well-adapted *Pgt* races to reproduce clonally and spread across large areas within relatively few years (Hermansen, 1968; Brown and Hovmøller, 2002; Hovmøller et al., 2016; Walter et al., 2016).

It may be difficult to establish the exact geographical or evolutionary origin of newly emerged genotypes/races of airborne plant pathogens due to lack of regular and extensive population surveys. In this study, we used a combination of molecular genotype and race phenotype assays, which were designed for resolving long-term genetic relationships using selective neutral markers, and short-term responses to host induced selection governed by R-genes in host plants, respectively. We observed evidence of resampling of some of the genotypes/races, which have been detected previously in Europe, for example in the local outbreaks in Germany (2013): Clade VIII (RFCNC), Clade IV-E.1 (TKKTF), and Clade IV-E.2 (TKKTF). Clade VIII was resampled several times in Czech Republic and Slovakia (race RFCNC) and in Hungary (race RFCPC). Otherwise, Clade VIII has been reported in a single isolate (race RSBNC) from Italy in 1985 (Szabo et al., 2022).

Clade III-B consisted of a single race (TTRTF) and five closely related MLGs and may designate the return of stem rust in Europe at epidemic levels (Bhattacharya, 2017). The most prevalent MLG and race within Clade III-B was prevalent in Eritrea in East Africa in the same year (Patpour et al., 2020), but the first observation dates back to 2014 in Georgia, where it was sampled at low frequency in a genetically diverse population (Olivera et al., 2019). Clade IV-F, which in this study contained a single race (TKKTF), was first detected in Europe in 2018, but earlier on detected in Georgia in 2014 in the southern Caucasus region (Olivera et al., 2019), and in 2015 and 2016 in Azerbaijan, Iraq and Eritrea (GRRC).^4^ Clade IV-B, which in this study was first detected in Croatia in 2017, became widespread in Western Europe between 2019 and 2021 (this study), and recently race TKTTF was confirmed in the United Kingdom and Ireland (Tsushima et al., 2022). Alignment efforts at GRRC using isolates from the latter study (Ireland: 2020; UK: 2019, 2021) confirmed the presence of Clade IV-B and race TKTTF in these countries. The same genetic group has been widespread in multiple countries in East Africa and the Middle East in previous years (GRRC), but in East Africa harboring two races, TKTTF and TTTTF (GRRC), suggesting a close relationship between these.

In contrast, race TKTTF was detected in three distinct but related genetic groups. In East Africa, TKTTF, also known as the ‘Digalu’ race, was assigned Clade IV-A.1 and Clade IV-B (Olivera et al., 2015), and in the German outbreak in 2013, TKTTF was represented in both Clade-A.1 and Clade-A.2 (Olivera Firpo et al., 2017). This may suggest independent evolution of virulence in these genetic groups, stressing that a combination of molecular genotype and race phenotype assays are crucial to resolve the population biology of biotrophic plant pathogens such as *Pgt*, where disease prevention is often governed by R-genes in the host plant.

In contrast to the clonal population structure in most areas investigated in this study, high levels of diversity with respect to SSR and virulence were detected in local areas in Spain, Sweden and western Siberia where alternate hosts for yellow- and stem rust, *Berberis vulgaris* and/or indigenous subspecies are present (Rodriguez-Algaba et al., 2021). The indicators of sexual reproduction in these local areas were among others, (i) high numbers of MLGs and races relative to sample sizes, (ii) high diversity indexes, (iii) low index of association (almost random association in several cases), and (iv) deficit or proportional levels of heterozygosity compared to expected under HW equilibrium. For the Spanish (barberry) and Swedish populations, the observed heterozygosity was lower than the expected indicating some degree of inbreeding in these local, diverse populations in proximity to the alternate host.

Several of the same features have been observed in sexually reproduced populations of *P. striiformis* on wheat in the Himalayan region of Asia (Ali et al., 2014; Hovmøller et al., 2016; Thach et al., 2016; Chen et al., 2021), oat and rye stem rust populations in Sweden (Berlin et al., 2012), wheat stem rust in Georgia (Olivera et al., 2019), and with respect to race distribution in local populations of wheat stem rust in the United States (Groth and Roelfs, 1982). The features above are strong indicators of *Berberis* spp. as an important component in the epidemiology of wheat stem rust in Europe, although unique genotypes/races from local populations in Sweden/Spain were rarely observed in distant areas. We observed a unique MLG and race in Denmark in 2020 that was first detected in a local sexual population in Sweden in 2019, and a unique genotype sampled in Norway in 2021 was also part of the sexual population in Sweden. Overall, our observations suggest that stem rust is persistent in Europe from one year to the next, and more intensive sampling efforts would most likely reveal additional cases of resampling of genotypes derived from sexual reproduction on *Berberis* spp. in Europe.

The study revealed highly interesting features with respect to race and virulence in the European population. It was striking that in most cases, only a single race was detected within an individual MLG, occasionally two races only differentiated by a single virulence character. The SSR genotyping therefore to a large extent reflects the presence of individual races, which represents a cheap and rapid complementary tool for time consuming and costly race assays. However, in a longer time and area perspective, additional virulences may emerge within existing genotypes, in stem rust most evident by the evolution of 15 races within the Ug99 genetic lineage [Singh et al. (2011); Patpour et al. (2015); RustTracker website], and in wheat yellow rust there are multiple examples Wellings and McIntosh (1990); Hovmøller et al. (2011); and Thach et al. (2016). The latter feature stresses the importance of a continuous search for additional genotyping markers and procedures in pathogen surveillance programs to keep pace with the ongoing evolution of new races.

*Sr31*-virulence was observed in multiple and highly diverse races in local populations in Spain, and a single case in a population from Siberia. None of these were related to Ug99, demonstrating that *Sr31*-virulence is not unique to Ug99 (Pretorius et al., 2000), which has been the general conception since the emergence of this historical race in East Africa in 1999 (Olivera et al., 2022). The latter study reported a single isolate carrying virulence to *Sr31* collected in 2018 in one of the local barberry areas in Spain, in proximity to the ones represented in our study. Twenty-two of the 28 recovered races from these areas carried *Sr31*-virulence in our study, irrespective of cereal/grass host origin, i.e., bread wheat, rye and a grass weed, *Elymus* spp. All isolates of these diverse races were successfully recovered and tested on wheat differential lines, which demonstrate that wheat adapted stem rust may also grow on rye, barley and wild grasses. These observations call for additional studies about existing and potential break-down of the host barriers that have been used to define *formae speciales* within *P. graminis* (Anikster, 1985). The presence of a *Sr31*-virulent race in an independent Siberian population stress that virulence may emerge independently and multiple times when large geographical areas and time spans are considered.

The present study was facilitated by long-term commitments of collaborators from leading rust diagnostic laboratories in Europe, United States and Russia. We demonstrated a successful alignment of two genotyping approaches and race phenotyping methodologies employed by different laboratories, which represents a major step forward in our efforts to strengthen coordinated rust surveillance for international research projects and global early warning systems for not only stem rust, but also yellow rust and leaf rust infecting wheat. Alignment and common standards for experimentation and exchange of data and results has made it possible to present new results from multiple laboratories in a global context, as demonstrated by the Wheat Rust Toolbox hosted by GRRC and associated websites for everyone interested. The burning question in years ahead is to which extent it will be possible to sustain the attention of both public and private donors to further develop even more timely and accurate early warning for disease outbreaks in major agricultural crops in Europe and beyond.

## Conclusion

The present study has several implications for plant health with respect to wheat production in Europe and beyond. The lack of stem rust resistance in widely grown European wheat varieties stress an urgent need to initiate new breeding efforts with a focus on stem rust resistance in current and future breeding programs, which could involve both conventional breeding methodologies (Singh et al., 2015) and use of new approaches (Luo et al., 2021; Wulff and Krattinger, 2022). The rapid recolonization of stem rust across large wheat growing areas, where the disease has been absent for decades, the documentation of recent exotic incursions of highly virulent races from areas outside Europe, and the existence of genetically diverse, local stem rust populations in proximity to the alternate host, *Berberis* spp., capable of infecting multiple host species such as barley, bread wheat, durum wheat, spelt, rye and grass weeds, stress the importance of regular and coordinated pathogen and disease surveillance efforts at both European and global scales. Epidemiological studies about similarities/differences in aggressiveness between isolates of prevalent clades and potential temperature adaptation of stem rust to cooler climates would also be highly relevant.

## Data Availability Statement

The original contributions presented in the study are included in the article/supplementary material. The SSR genotypic data of the 459 isolates are available at https://doi.org/10.5281/zenodo.6598616.

## Author Contributions

MH, MP, JR-A, and AJ designed the study and wrote the manuscript. MP recovered and multiplied live isolates and performed the race phenotyping. JR-A, TT, and AJ performed the genotyping and data analyses. LS performed the SNP genotyping of reference isolates at CDL. AB, YJ, and DV designed sampling strategy and facilitated sampling in barberry areas in Sweden and Spain, respectively. VS and ES designed sampling strategy and facilitated sampling in Western Siberia. BR, KF, PC, AH, SS, KM, and RV facilitated sampling in Italy, Germany, Poland, Czechia, Slovakia and France, respectively, and ensured alignment with race phenotyping results in the national rust surveillance programs in these countries. JH uploaded genotyping and phenotyping data to the Wheat Rust Toolbox and developed the graphical presentation of results on maps. All authors contributed to the article and approved the submitted version.

## Funding

Major part of this work was supported by the European Union’s Horizon 2020 research and innovation programme under grant agreement no. 773311 (RustWatch), long-term alignment efforts across genotyping platforms (SNP and SSR) conducted by the Cereal Disease Lab and Aarhus University (GRRC), and rust sampling in proximity to *Berberis* spp. in Spain were supported by Delivering Genetic Gain in Wheat project supported by the Bill and Melinda Gates Foundation, the UK Department for International Development, and Innovation Fund Denmark, Ministry of Higher Education and Science (grant no. 19052, MULTIRES). Sampling and characterization of samples from Sweden 2017–2018 was supported by grants from FORMAS (2017-02311) and Jordbruksverket (2018-02-27).

## Conflict of Interest

The authors declare that the research was conducted in the absence of any commercial or financial relationships that could be construed as a potential conflict of interest.

## Publisher’s Note

All claims expressed in this article are solely those of the authors and do not necessarily represent those of their affiliated organizations, or those of the publisher, the editors and the reviewers. Any product that may be evaluated in this article, or claim that may be made by its manufacturer, is not guaranteed or endorsed by the publisher.

## Footnotes

^1^ http://agro.au.dk/forskning/projekter/rustwatch/

^2^ www.wheatrust.org

^3^ www.wheatrust.org

^4^ www.wheatrust.org
