# Supplementary material for: Wheat Stem Rust Back in Europe: Diversity, Prevalence and Impact on Host Resistance
Source: Front Plant Sci. 2022 Jun 2;13:882440. doi: 10.3389/fpls.2022.882440 (PMC9202592; doi:10.3389/fpls.2022.882440)
Supplement: Supplementary file 1 [file Data_Sheet_1.PDF]

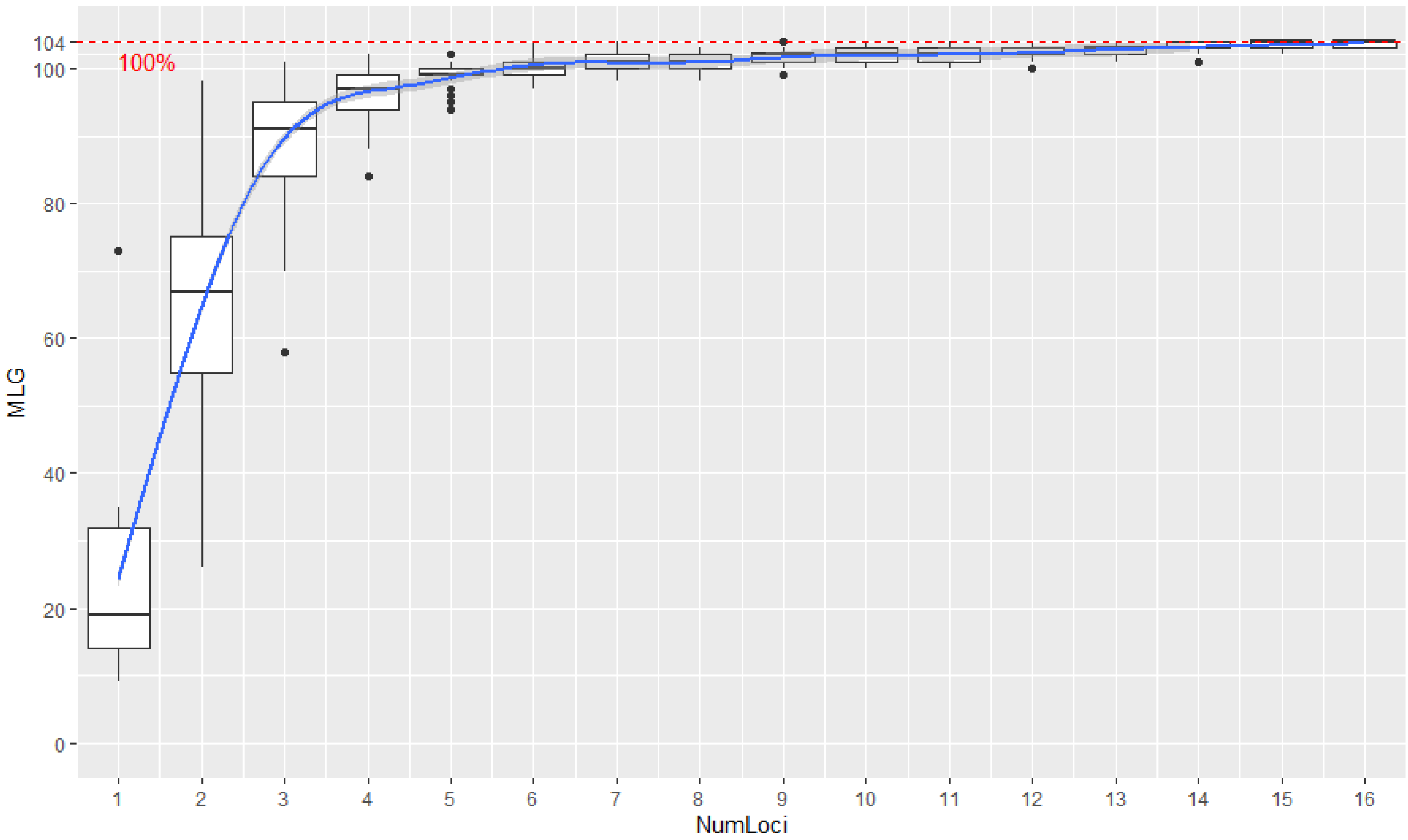

Figure S1. Multilocus genotype resolution by 17 SSR primer pairs used to study genotypic diversity in *P. graminis* f.sp. *tritici* in Europe. The full curve represents maximum resolution with the SSR loci and 104 distinct MLGs including samples, where missing data for a single locus were allowed.

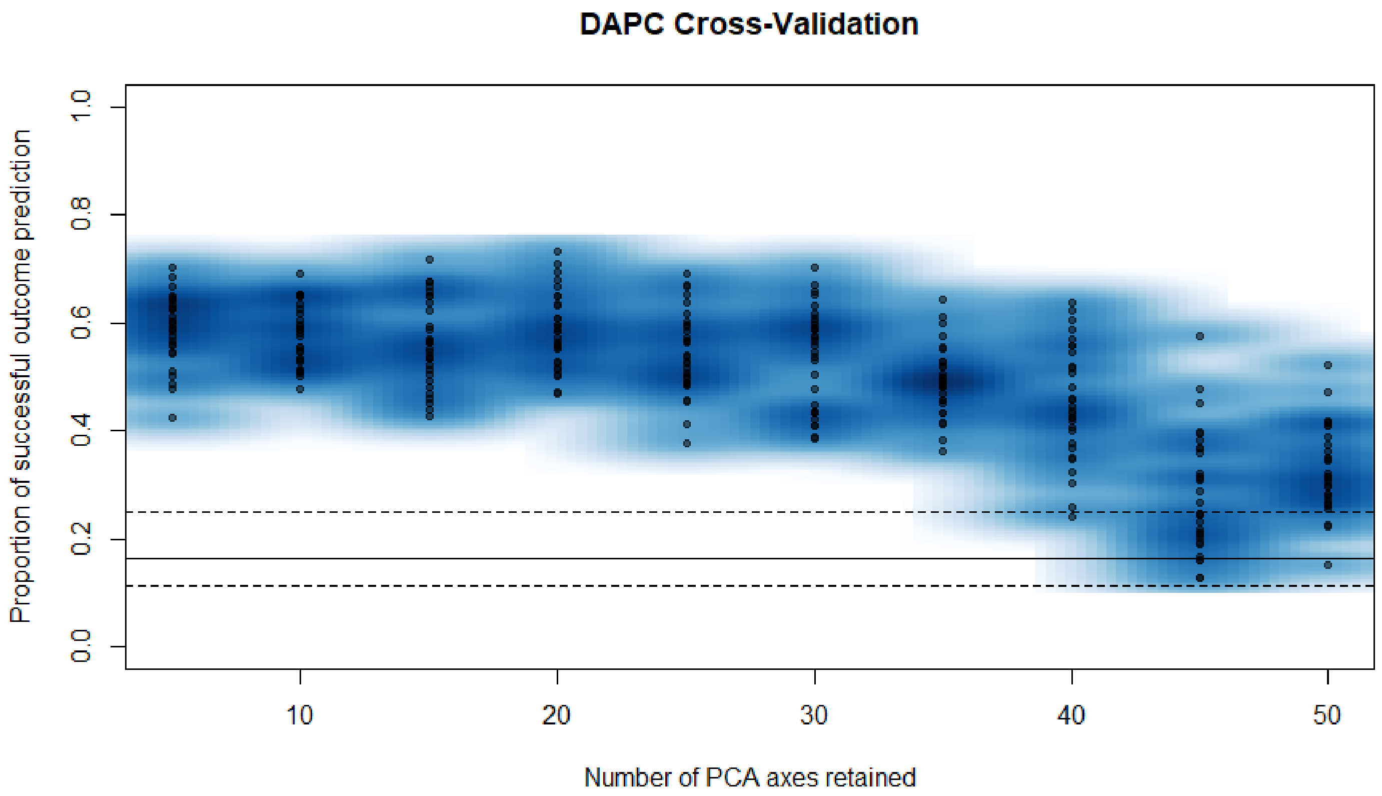

Figure S2. Cross-validation of the discriminate analyses of principle components (DAPC). Each dot represents individual replicates, which supported 20 PCAs to be retained.

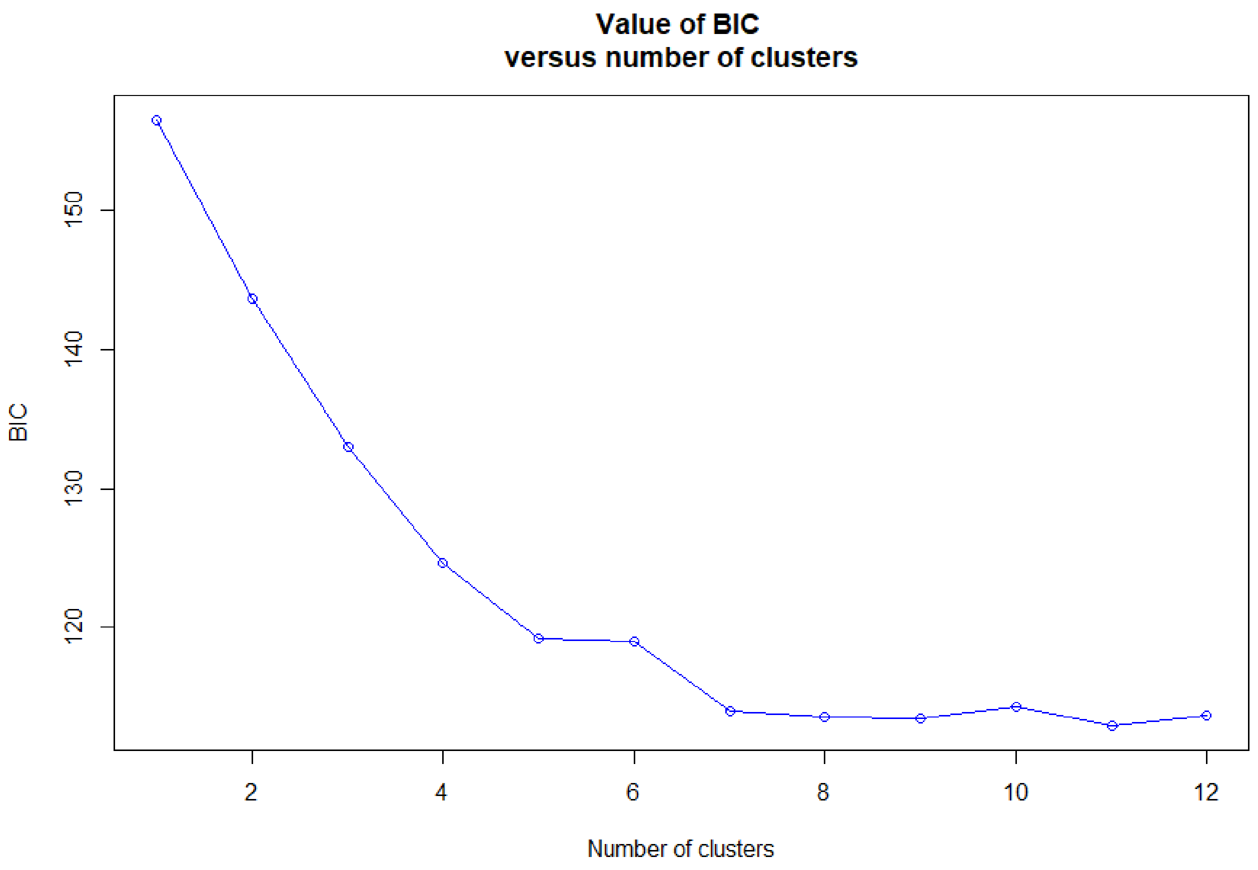

Figure S3. Bayesian information criteria (BIC) plotted against the number of clusters in the discriminate analyses of principle components (DAPC). The BIC supported five distinct genetic clusters.
